# Toward model-based individualized fitting of hip-flexion exosuits for persons with unilateral transfemoral amputation

**DOI:** 10.1017/wtc.2025.5

**Published:** 2025-03-12

**Authors:** Finn G. Eagen, Nicholas P. Fey

**Affiliations:** 1 Walker Department of Mechanical Engineering, The University of Texas at Austin, Austin, TX, USA; 2 Texas Robotics Consortium, The University of Texas at Austin, Austin, TX, USA

**Keywords:** biomechanics, exosuits, design, gait, optimization

## Abstract

The muscular restructuring and loss of function that occurs during a transfemoral amputation surgery has a great impact on the gait and mobility of the individual. The hip of the residual limb adopts a number of functional roles that would previously be controlled by lower joints. In the absence of active plantar flexors, swing initiation must be achieved through an increased hip flexion moment. The high activity of the residual limb is a major contributor to the discomfort and fatigue experienced by individuals with transfemoral amputations during walking. In other patient populations, both passive and active hip exosuits have been shown to positively affect gait mechanics. We believe an exosuit configured to aid with hip flexion could be well applied to individuals with transfemoral amputation. In this article, we model the effects of such a device during whole-body, subject-specific kinematic simulations of level ground walking. The device is simulated for 18 individuals of K2 and K3 Medicare functional classification levels. A user-specific device profile is generated via a three-axis moment-matching optimization using an interior-point algorithm. We employ two related cost functions that reflect an active and passive form of the device. We hypothesized that the optimal device configuration would be highly variable across subjects but that variance within mobility groups would be lower. From the results, we partially accept this hypothesis, as some parameters had high variance across subjects. However, variance did not consistently trend down when dividing into mobility groups, highlighting the need for user-specific design.

## 1. Introduction

A transfemoral amputation (TFA) is a major surgery with life-altering effects (Behera and Dash, 2021). The knee and ankle joints are each essential for gait and balance, and the loss of both has a significant negative impact on the mobility of the individual (Helm et al., 1986; Davies and Datta, 2003). Preservation of mobility is important as it has a strong effect on quality of life. High mobility allows individuals to more readily participate in their community and attend to their personal health needs, whereas low mobility often leads to secondary disorders such as abnormal weight gain and depression (Fortington et al., 2012; Chopra et al., 2018). Even in the best of cases, individuals who have undergone a TFA will likely never be able to walk with the same ease as an able-bodied person. An average individual with a unilateral TFA will perform roughly 1500 steps in a day, versus 8500 for a comparable able-bodied individual (Miller and Brown, 2004; Halsne et al., 2013). It has been well-studied that further physical therapy and more advanced assistive devices both aid in raising the mobility of an individual with a lower limb amputation (LLA) (Wong et al., 2016; Ülger et al., 2018; Gailey et al., 2021; Tran et al., 2022; Simon et al., 2023). However, such means are unfortunately inaccessible to most individuals who have experienced LLA. Physical therapy is costly, time-consuming, and fatiguing. Few have the additional funds and energy to do more than is prescribed. Simultaneously, advanced assistive devices like powered joints have limited commercial availability and are not covered by many insurance providers due to their high cost. For both of these reasons, many individuals with an LLA must settle for suboptimal care.

In the U.S., device accessibility is primarily determined by Medicare functional classification levels, known as K-levels. Ranging from 0 to 4, K-levels theoretically capture the full range of functional outcomes for individuals with LLA. K0 is described as an individual who cannot safely use a prosthesis, whereas K4 is someone who “exceeds basic ambulation skills,” such as athletes (Gailey et al., 2002). K-levels have been heavily critiqued, given that they are a largely subjective and very coarse system (Borrenpohl et al., 2016). Despite this, they are the most common classifier used for individuals with LLA, making it a necessary lens through which we explore the experiences of individuals with LLA. The largest body of individuals, those in K2 and K3, can walk for short distances without assistance, though often find the task difficult and fatiguing (Gailey et al., 2002). Such individuals are likely to develop secondary disorders due to mobility issues, and yet, due to their K-level, do not have access to devices that could increase their mobility. Under Medicare, neither K2 nor K3 individuals have coverage for motorized prostheses, and K2 individuals are additionally not covered to access microprocessor-controlled knees except in special cases (Medicare Coverage Database, 2025). These barriers still exist despite the fact that both devices have been shown to improve the mobility of K2 and K3 users (Hafner and Smith, 2009; Simon et al., 2023). This leaves K2 and K3 individuals with few options for further care. As such, this work is centered around increasing the accessibility of assistive devices for such individuals. We chose to specifically target the hip of the residual limb as this joint plays a major role during gait and is often unassisted.

For individuals with a TFA, the muscles of the hip must be retrained to adopt the prior functions of lost joints. For instance, swing initiation, previously performed by the plantar flexors, is achieved through over-activating the hip flexors (as compared to able-bodied gait as a baseline). This is in addition to other compensatory mechanisms related to forward propulsion and balance management (Wentink et al., 2013). Altogether, the higher muscle activity in the residual hip is a major factor that makes walking a more strenuous and fatiguing task for individuals with TFA (Harandi et al., 2020). Ample research has gone toward the development of advanced prosthetic feet, ankles, and knees in an effort to mitigate this strain, often to great success (Azocar et al., 2018; Elery et al., 2020; Tran et al., 2022). However, there has been less development toward direct assistance of the hip—specifically low-cost, accessible options. Multiple projects have applied passive hip exosuits for gait correction and assistance, though for patient populations other than those with TFA. Neuman et al. applied a two-band hip flexion orthosis to assist swing initiation and correct gait asymmetry in individuals with multiple sclerosis (MS) (Neuman et al., 2021). Similarly, Kowalczyk et al. have shown that a two-band device mounted across the back of the hip was able to correct artificial gait asymmetry in able-bodied individuals (Kowalczyk et al., 2023). In both cases, a low force, low profile, and low-cost device was able to significantly affect and ease the gait of the subjects. Compared to passive hip assistance, active hip exoskeletons have seen much more development (Panizzolo et al., 2019; Zhang et al., 2022). Particularly, the work of Ishmael et al., which aided individuals with TFA showed major positive effects on the metabolic cost of transport (Ishmael et al., 2021). Unfortunately, active hip exoskeletons face similar issues as active prostheses when it comes to accessibility. Limited commercial availability, lack of insurance coverage, and device limitations in battery size and weight have prevented hip exoskeletons from yet being integrated into the standard of care (Schiele, 2009; Zhang et al., 2017).

In this work, we put forth two contributions on the topic of direct hip assistance for individuals with TFA. Both explore the potential impact, through simulation, of a wearable device for hip assistance. The first is the novel application of a passive hip flexion exosuit. Given the success of similar devices with other patient populations, we believe such a device will be highly successful in reducing fatigue for individuals with TFA. This low-cost solution will make hip assistance available to a wide range of users, regardless of mobility classification. The second contribution employs the same simulation methods to optimize control variables for an imagined cable-driven motorized exoskeleton. We are interested in exploring the feasibility of a wrapping, cable-driven hip flexion exosuit, as it allows for more versatile and discrete form factors, reducing one of the barriers of acceptance with active devices. However, if and when the force-generating element is not aligned with the biological joint, the control of each cable becomes a more nuanced problem. Much like the proposed passive exosuit made of elastic elements, the behavior of the device becomes dependent on the user-specific wrapping of each cable. Prior to applying either device in human subject experiments, we sought to develop a simulation framework for the initial optimization of device parameters. Human-in-the-loop (HIL) optimization is a time-consuming and fatiguing task, especially for individuals with gait difficulties. The simulation methods proposed here will inform future experiments, reducing the time necessary with human subjects. In this article, we model the behavior of a wrapping assistive hip flexion device for 18 individuals with TFA, 9 of K2 and 9 of K3 classifications (Hood et al., 2020). For each subject, we solve for an optimal parametrization of the device with respect to two different cost functions, reflective of a passive and active implementation. We hypothesize that for both of these implementations, the optimal configuration of the device will have a high variance between subjects but that distinguishing the results by K-level will reduce the variance.

## 2. Methods

### 2.1. Creating the model

**Figure 1.**
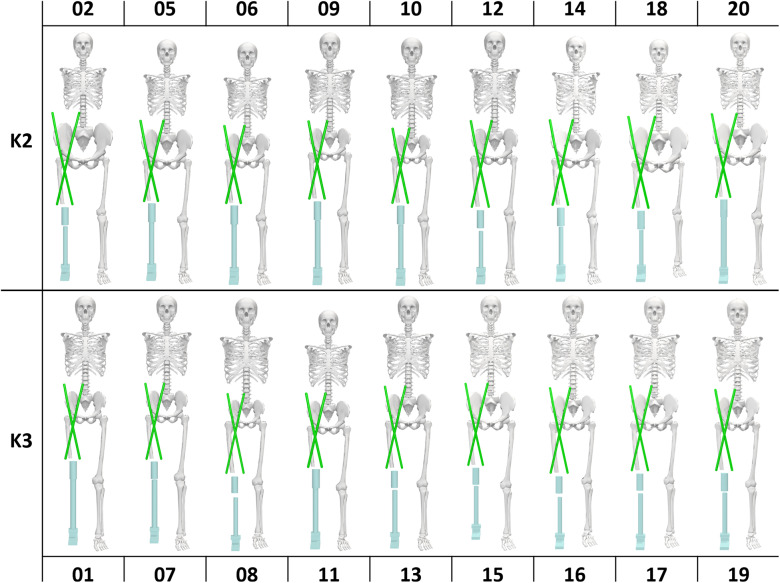
Uniquely scaled models for each of the 18 subjects, numbered by the identifier given in the Hood et al. dataset. The subjects are organized by K group, with K2 on the top row and K3 on the bottom row.

We first created an OpenSim musculoskeletal model of an individual with a TFA and prosthesis (Delp et al., 2007). Using the model of an individual with TFA created by Ranz et al. as a base, we added segments for the prosthetic shank and foot of the amputated limb (Ranz et al., 2017). The model was then scaled with respect to the subject’s weight and proportions according to data provided by Hood et al. The dataset also provided the exact knee and foot prosthesis used by each individual, allowing the mass and approximate inertial properties of each device to be prescribed during the scaling process (Hood et al., 2020). Following scaling, we had 18 unique models that accurately represented the shape and size of each subject, as can be seen in Figure 1.

Each model also included a wrapping surface about the pelvis. The behavior of the device is heavily dependent on the wrapping of the elastic elements over the hip. The modeling work of Neuman et al. illustrates this importance via a robust wrapping calculation that considers a deformable human shape (Neuman and Fey, 2023). OpenSim natively contains a wrapping algorithm, though it is limited to a few primitive shapes. Notably, the wrapping calculation done by OpenSim is prone to discontinuities when dealing with more than one surface. Despite its flaws, we chose to work with OpenSim’s native wrapping surfaces. Compared to relying on an external wrapping calculation, performing the calculation in OpenSim allowed for more streamlined analysis, as well as notably leaving the model easily transferable to OpenSim Moco and OpenCap (Dembia et al., 2020; Uhlrich et al., 2023). To ensure continuity, we chose to use a single ellipsoid mounted at the pelvis. This came at the sacrifice of accuracy in our wrapping calculation; however, we believe it is still an effective representation of the wrapping.

The device itself was modeled as two flexible linear elements, each wrapping over the previously mentioned surface. Each band has an origin located on the waist and insertions just above the knee. The bands are crossed to allow for balance in the rotation axis. This design was inspired by Neuman et al. and Kowalczyk et al., which both employed crossed-band designs (Neuman et al., 2021; Kowalczyk et al., 2023). These origin and insertion points were fixed across all subjects. The elements were modeled as linear springs, each with a stiffness and resting length.

### 2.2. Simulation

Having created 18 unique subject models, we then performed analyses to determine joint angles and moments for an average stride of each individual at 0.8 m/s over level ground. Of the speeds provided in the dataset of Hood et al., we chose to use the trials at 0.8 m/s as this speed was available for both K2 and K3 individuals and would allow for an effective comparison between the groups (Hood et al., 2020). While the dataset does supply joint moment values for the sagittal plane, we sought to analyze the effect of the device in all three axes of the hip. This necessitated that we perform the analysis ourselves. Motion capture marker data were used to create a skeletal model in Vicon Nexus (Vicon, Oxford, UK), and the resulting motions were input to OpenSim. This includes scaling the OpenSim model appropriately, as well as running inverse kinematics (IK). The joint angles provided by IK were then combined with the ground reaction force (GRF) values provided by the dataset to perform inverse dynamics (ID) calculations, returning joint moments over the course of each stride. This analysis was performed for all strides of each subject (roughly 45 strides per subject). Across all subjects, 4 strides had to be excluded due to being extreme outliers. Each individual stride was then separated, and an average stride was computed for each subject. This average stride contains both the average joint angles and the average joint moments. As the device model is integrated into the musculoskeletal model, we could then easily retrieve the length and moment arms of each band over the course of the average stride.

For the scope of this work, the kinematics were assumed to be fixed, that is, the device would not have an impact on the gait pattern of the individual. This simplifying assumption significantly reduces computational load as it limits the work to non-predictive simulations. An assumption of fixed or enforced kinematics is not unreasonable for optimization of this sort and has commonly been used in similar works (Boĺivar et al., 2017; Dembia et al., 2017; Neuman and Fey, 2023). Moreover, the human subject experiment of Neuman et al. shows this assumption holds in able-bodied users for passive configurations of the particular device we consider (Neuman et al., 2021). However, we fully acknowledge the negative impact of fixed kinematics on the validity of the results. For this reason, we do not suggest the methods that follow will provide immediate optimal results in a physical realization. Though the simulation still holds value in providing an informed initial parametrization that will ideally reduce the length of HIL experiments. Additionally, within the bounds of the method, we can extract valuable relationships between subject characteristics and outcomes.

### 2.3. Optimization

**Figure 2.**
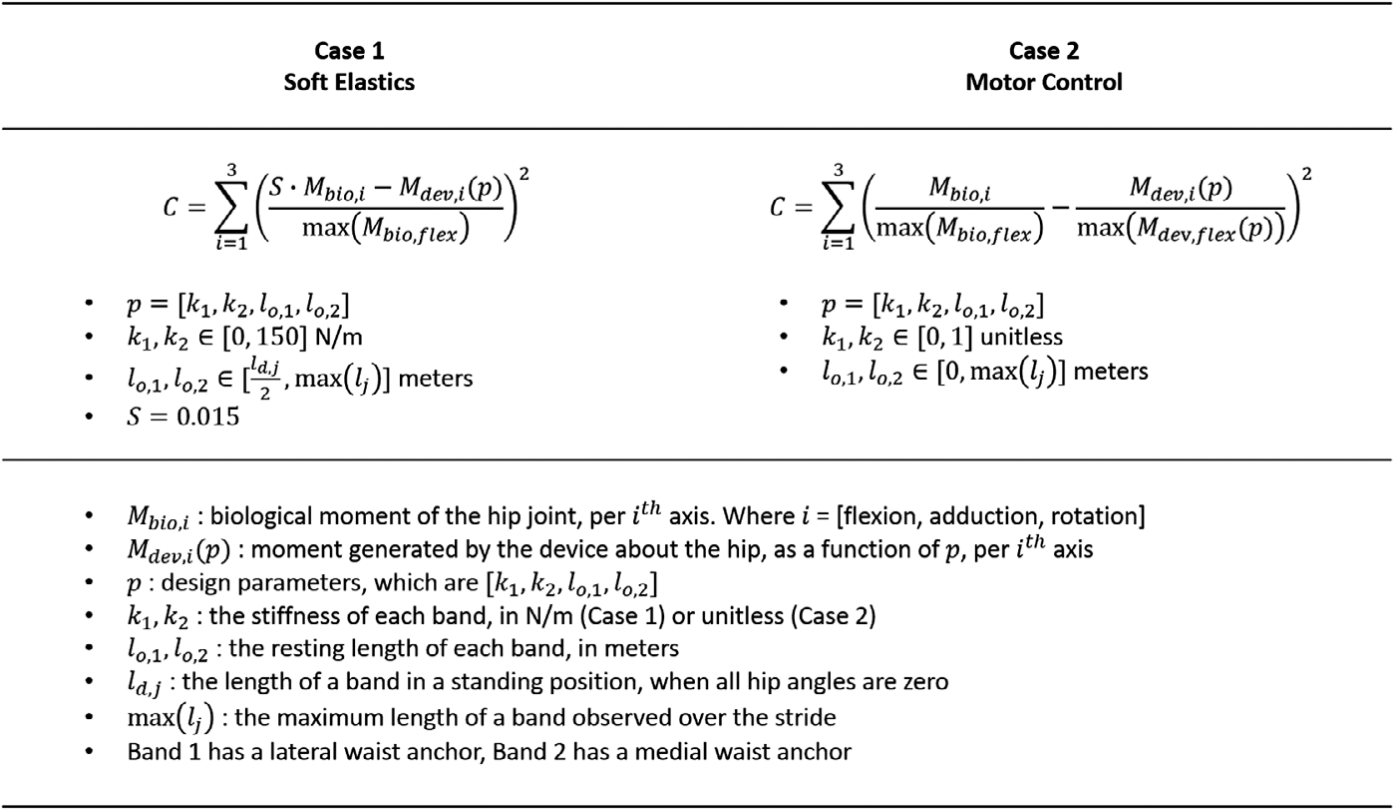
Graphic describing the cost function used for each case, along with the bounds on each parameter. Below the bounds is a listing of all the variables used in the functions.

Having determined the geometric behavior of the device over the course of the average stride for each individual, we sought to determine a unique, optimal device configuration for each user. The optimizations consider the resting length and stiffness of each band, a total of four parameters. For the two devices considered in this work, we propose two similar cost functions, described in Figure 2. For both cases, the cost function is minimized via the interior-point algorithm. The optimizer averaged roughly 2 minutes to find a solution for a given subject and cost function.

The first function, Case 1, is built to return results that are reasonable for a soft elastic device. Here we take the difference between 1.5% of the biological moments and the moments generated by the device. This difference is normalized by the maximum biological moment in the flexion direction and squared, then summed over all three axes. This cost function is minimized when the device well matches 1.5% of the biological moment in all three axes, which is our desired behavior. In this first case, the stiffness of each band is bounded to a maximum of 150 N/m, and the resting length of each band can be no shorter than half of its span in a standing position. Shorter bands pose a feasibility concern as pre-tensioning the device while donning would become a cumbersome task, while the limit on stiffness is reflective of a reasonable upper bound on the stiffness of a flexible resistance band. The scaling factor of 1.5% was chosen as this was the greatest value that returned consistent results under the imposed bounds. Setting a higher goal resulted in degenerative cases where the optimal configuration was simply the parameter set that generated the most force. This outcome would obscure differences between subjects and was not meaningful to analyze. We progressively lowered the goal until all subjects avoided the degenerative case, which was successful at 1.5%.

Case 2 is built to highlight the application of these methods for motor control. The cost function operates similarly to that of Case 1; however, now the biological moment and device moment are each normalized by their respective maximums in flexion. In effect, this computes the squared difference of the shape or profile of each moment. This approach is device agnostic as it does not consider the absolute force generated by the device, which would depend on the choice of components. This method could account for any control law, though in this work, we consider a straightforward controller that simulates a linear spring along each element. We solve for unitless “stiffness” values, which can be understood as relative motor gains, and “resting lengths” that are as low as zero, no longer bounded by physical constraints. The important consideration is that by using these proposed methods, which simultaneously account for the moment generated in all three axes of the hip, we solve for motor control parameters that consider to the user-specific wrapping of the elements.

## 3. Results

**Figure 3.**
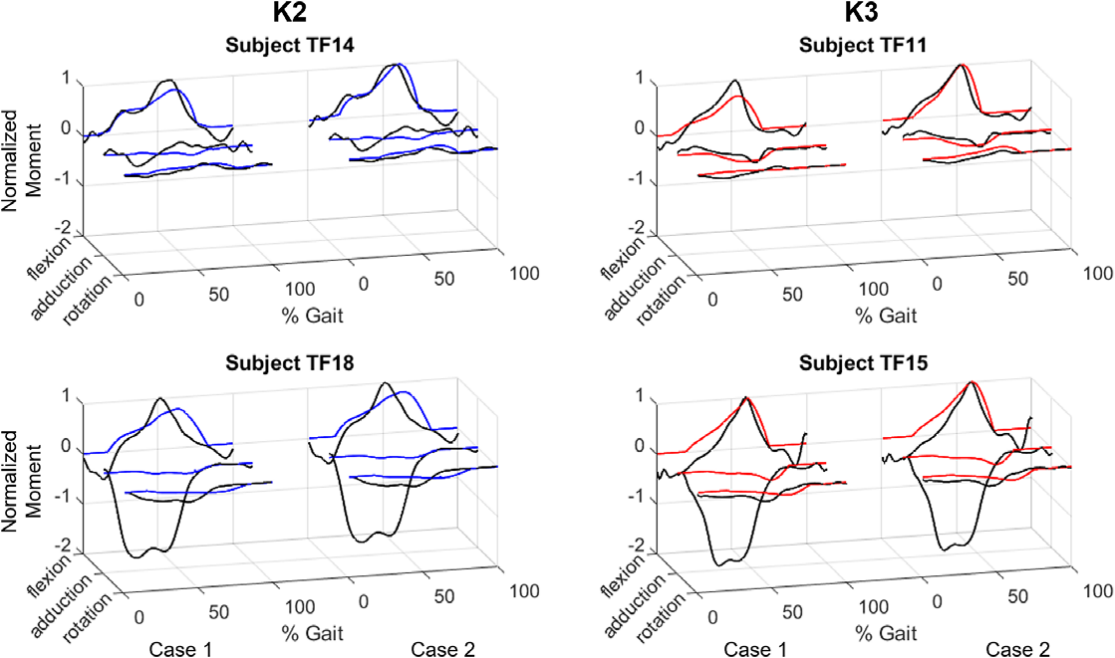
The optimizer results for four representative subjects. Two subjects were chosen from each group (K2 in blue, K3 in red), with the lowest RMSE of a group on the top row and the highest RMSE on the bottom row. The results are shown for both cases (Case 1: Soft Elastics (left), Case 2: Motor Control (right)). The biological moment is shown in black, and the device moment is shown in color. The cases are normalized to compare the relative quality of each match.

**Figure 4.**
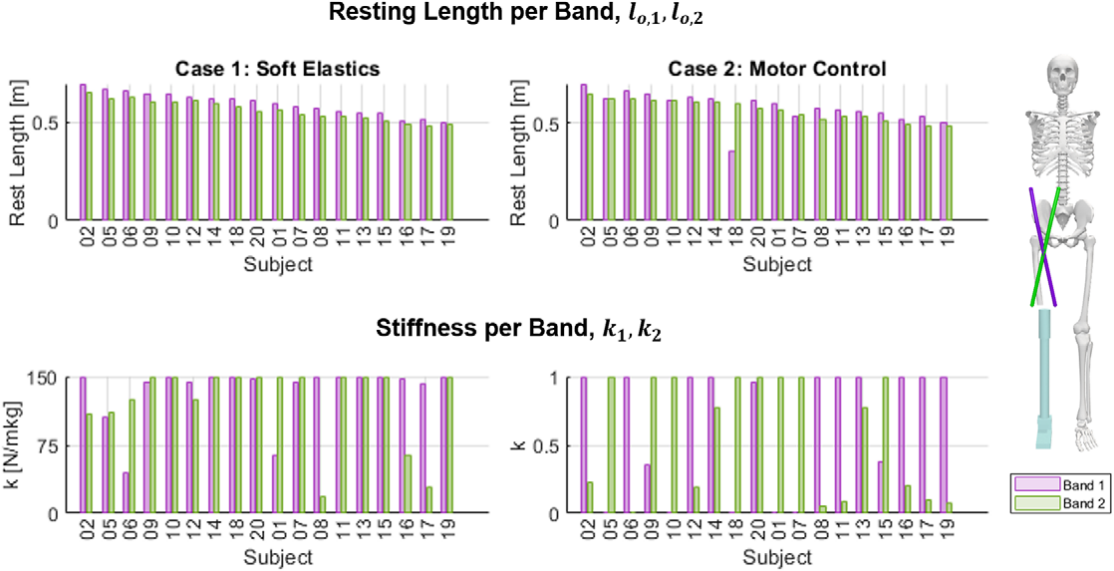
The stiffness and resting length of each band for each subject across both cases. The values for Band 1 and 2 are shown in purple and green, respectively. A reference model is included to help identify each band. Subjects are ordered by their average band resting length.

The optimization found a unique device profile for each individual in both cost functions. The moment profiles for a select number of subjects are shown in Figure 3. The fit is shown for each cost function and split into each axis of the hip. In Figure 1, we report the mean value and variance of optimization parameters and success metrics across all 18 subjects as well as within each K group. The optimal device properties are shown for each subject in Figure 4.

For Case 1, representative of a passive device, root mean square error (RMSE) across all 18 subjects had a mean value of 0.277 and a variance of 0.023. The average achieved peak compensation was 1.156%, compared to a goal percentage of 1.5%. Variance, however, was low at >0.001%. We also report mean percentage error (MPE). Compared to RMSE, MPE places a higher value on the error when the biological moment is small. Additionally, as a signed value, it indicates whether the device is generally under or overcompensating. For the whole group, the mean MPE in Case 1 was 159.76%. For the optimization parameters, the mean resting length of each band was 0.562 and 0.595 m, respectively, with a low variance of 0.003 m for each. The stiffness of each band had an average of 123.8 and 135.1 N/m each, with variances of 1757.9 and 934.4 N/m. Recall that the stiffness for this case was bounded to a maximum of 150 N/m. In Case 2, fit for motor control of a cable-driven device, RMSE across the whole group had a mean value of 0.271 and a variance of 0.022. This average RMSE is slightly lower than the average of 0.277 for Case 1. By contrast, the mean MPE value was significantly higher than Case 1 at 284.63%. The average simulated rest length of each band was 0.568 and 0.582 m, with low variances of 0.003 and 0.006 m. The normalized stiffness values of each band were 0.581 and 0.650, with variances of 0.182 and 0.200, respectively.

Splitting the results of each method into K2 and K3 groups, the performance and average values diverged. Within K2 the RMSE for each case dropped to 0.242 and 0.238, with variances also decreasing to 0.020 for both. The mean percentile compensation in Case 1 lowered slightly to 1.140%. For MPE, it decreased significantly in both cases, to −23.93% for Case 1 and 12.90% for Case 2. The resting length of the bands in both Case 1 and Case 2 did not change greatly, though all decreased roughly 1 cm from the total average. For stiffnesses, the averages for Band 1 decreased by about 20%. The stiffness of Band 2 in Case 1 changed by less than 2 N/m, though in Case 2, the stiffness rose by 19%. Of note, the variance of the stiffness of each band rose significantly for Case 1 but decreased in Case 2.

It follows that within the K3 group, the parameters experienced mirrored changes. The RMSE for each case rose to 0.312 and 0.305, with a variance of 0.023 for each. Peak percentile compensation in Case 1 rose to 1.171%. MPE rose to 343.45% for Case 1 and 556.37% for Case 2. Resting lengths increased slightly, though within 2 cm of the whole group average. The stiffness of Band 1 increased by roughly 20% for both cases, while the stiffness of Band 2 was nearly unchanged in Case 1 but decreased in Case 2 to 0.525. The variance for both stiffnesses in each case decreased, though the most drastic change was the stiffness of Band 1 in Case 1, which fell to 190.2 N/m. Variances did not trend uniformly in either case when splitting the results into K2 and K3 groups. Additionally, the graphs in Figure 4 clearly show that the high variances were not due to a small set of outliers, but rather that results consistently varied across all subjects.

In addition to computing the mean and variance of each parameter, we also sought to test for interdependence of the optimization variables. We achieved this by computing Pearson’s correlation coefficient for each parameter over all 18 subjects. By checking for correlation, we hoped to rule out the possibility that any parameter was easily derived from another. In this test, only two sets had a coefficient stronger than ±0.6. The first was the resting length of Band 1 and Band 2 in Case 1, these variables had a strong coefficient of 0.98 (*p* < 0.001). This correlation can be expected as an imbalance between the bands would lead to a net rotational moment. The second was the stiffnesses of Band 1 and Band 2 in Case 2. These had a weaker but still strong coefficient of −0.76 (*p* < 0.001). This negative correlation highlights the frequency with which the optimizer heavily biased one band, as is visible in Figure 4. Of note, the coefficients for relationships between any given resting length and any stiffness were all weaker than ±0.35, with *p* > 0.05, meaning that except for the examples discussed, variables were generally not strongly correlated.

**Figure 5.**
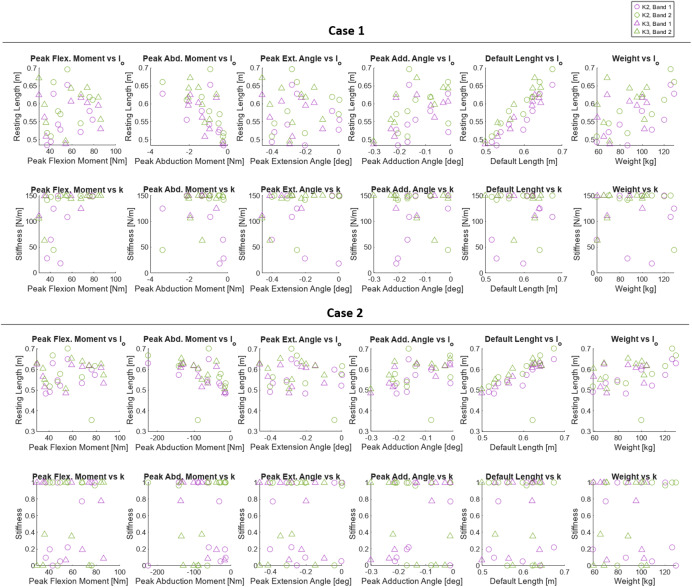
Correlations between various subject-specific characteristics and optimal parameters. For each Case, the top row is plotted against resting lengths, while the bottom row is stiffnesses. Each column of graphs is a subject characteristic. From left to right: peak flexion moment, peak abduction moment, peak extension angle, peak adduction angle, default length, and weight. “Default length” is the length of each band in a standing position. Purple is for Band 1, and green is for Band 2, as in Figure 4. Circles are for K2, whereas triangles are for K3 subjects.

Having retrieved individualized parameters for each user, we sought to determine if any particular subject characteristic could be used to inform or predict a user’s ideal fit. For this, we considered peak moments in the flexion and abduction directions, peak joint angles in extension and adduction, the default length of the bands, and the weight of the individual. The default length of the bands refers to the length of each band when all hip angles are zero. This parameter is largely a function of the length of the subject’s thigh, though it is also affected by the wrapping of the bands and the size of the pelvis. For each of these characteristics, we plot them against the design parameters for each case in Figure 5. Below the plots, we list the correlation coefficient and significance for each relationship, sorted by significance in the combined set in Table 2. In Case 1, the significant (*p* ≤ 0.05) correlations we observe in the combined set are default length to resting length (0.91, *p* < 0.001), weight to resting length (0.53, *p* = 0.001), peak abduction moment to resting length (−0.51, *p* = 0.002), peak adduction angle to resting length (0.49, *p* = 0.002), and peak flexion moment to stiffness (0.45, *p* = 0.006). Peak abduction moment to stiffness was significant in Band 1 (0.59, *p* = 0.010) but not in the combined group. In Case 2, relationships were largely band-specific and less significant. While multiple were significant at the combined level, the only relationship that held independent significance in both bands and combined significance was default length to resting length (0.69, *p* < 0.001). In Band 1, we found significant correlation in weight to resting length (0.53, *p* = 0.022), peak abduction moment to resting length (−0.61, *p* = 0.007), peak adduction angle to resting length (0.58, *p* = 0.012), peak abduction moment to stiffness (−0.56, *p* = 0.016), and peak adduction angle to stiffness (0.49, *p* = 0.038). For brevity, we have reported these values across the whole group of subjects, though we note that these results did not vary substantially when splitting into K2 and K3 groups.

## 4. Discussion

In this work, we consider the application of wrapping hip flexion assistive devices for individuals with a TFA. Through a simulation framework, we sought to optimize the potential impact of both a passive hip orthosis and an active cable-driven exosuit. Both applications share a model, created in OpenSim, which is illustrated in Figure 1. We first observe the physical behavior of the device during subject-specific level ground walking kinematics. Based on the device’s response to the unique shape and kinematics of each user, we then optimize over two cost functions, which respectively represent the passive and active forms of the device. For the passive device, the optimization is built to return resting lengths and stiffnesses that could be reasonably realized via soft elastic elements. For an active device, we solve for motor control parameters that simulate linear elastic elements, though no longer bounded by physical constraints. The aim of this work is to showcase the utility of force-generating elements that wrap about the hip, generating a moment in multiple axes simultaneously. The results suggest this device structure is viable for both passive and active forms. Even with only two force-generating elements in fixed positions, we are able to match the moment profile in each axis to a low RMSE for many subjects. The results were not uniform, however, as Figure 3 highlights the varied quality of results. Subject TF11 was well matched in all three axes, whereas Subjects TF18 and TF15 both had a major adduction moment the device was unable to account for. This difference in quality is also reflected in the MPE values, which highlight that, particularly in Case 2, there was a much greater presence of mistimed assistance. If band position was included as an optimization parameter, it is possible some more drastic moments could have been accounted for; however, even in its current state, the device provides well-timed assistance to the majority of users.

As we consider the two applications of our methods, the passive form of the device is of particular interest given the low cost to produce such a device. Due to the numerous complications regarding the availability and user acceptance of motorized devices, there are many instances in which a passive device is a far more reasonable means of assistance. Comparable devices have been well accepted as the standard of care for other gait differences and continue to see advancement due to their benefit of feasibility. Ankle–foot orthoses have been well applied to many patient populations to assist plantar flexor function, including those with cerebral palsy, individuals who have experienced lower limb trauma, and those poststroke (Lam et al., 2005; Harper et al., 2014; Skigen et al., 2024). Passive knee exoskeletons are less common devices, though they have been shown to reduce the metabolic cost of walking (Elliott et al., 2014; Etenzi et al., 2020). Specific to individuals with TFA, the advancement of passive prosthetic feet has shown major positive effects on the gait of users (Fey et al., 2011; Major et al., 2014). Yet, direct assistance of the hip via passive structures has been a less explored topic, specifically for those with TFA. As discussed previously, the work of Kowalczyk et al. and Neuman et al. has shown promise for such a device. Their work provides a firm base to suggest that a wearable device made of soft elastic elements could have a positive effect on the gait of individuals with TFA. While Neuman et al. do not report the stiffness of their device, the optimal parameters returned by our simulation are in line with the device of Kowalczyk et al. Their exosuit was estimated to provide 6.9 ± 1.4 N of force at full extension, compared with an average force of 7.8 N and a standard deviation of 1.6 N for our device (Neuman et al., 2021; Kowalczyk et al., 2023).

Compared to passive hip assistance, prior work toward motorized hip assistance is vast and diverse. The Composite Hip Exo at the University of Utah well showcases the effectiveness of direct hip assistance for individuals with a unilateral TFA (Ishmael et al., 2021). The exo was able to improve walking economy by 15.6% with only 10–15 Nm of peak torque. Relevant to the methods presented here, cable-driven devices have often been explored as an alternative to traditional exoskeletons. The freedom to place actuators far from the affected joint can be leveraged to reduce the inertial mass of the device and thereby reduce the cost of transport, or more simply, allow freedom in the form of the device to avoid interference with other wearables. The untethered cable-driven exosuit of Panizzolo et al. was able to reduce the metabolic cost of transport during a load-bearing task via assisting hip extension (Panizzolo et al., 2019). Cable-driven exosuits have also been well applied to the ankle, with the untethered system by Asbeck et al. reducing the metabolic cost of transport via aiding plantar flexion (Asbeck et al., 2013). A number of tethered devices have shown similar results, notably the exoskeleton emulator by Bryan et al., which aids the hip, knee, and ankle (Bryan et al., 2021). The devices mentioned, however, only employ a single actuator per joint, with strict and sometimes intrusive wire guides. As shown in this study, there is a possible benefit in the use of actuators purposefully aligned across multiple axes of a joint. By optimizing this behavior, we can generate beneficial moments in multiple axes with the use of fewer actuators. The possibilities only increase if we are to include the positioning of the band in the optimization.

We acknowledge the limitations of the presented methods. As is, the primary limitation is the assumption of fixed kinematics. The behavior of the device is dependent on the exact kinematics of the user, though in a fixed simulation, we do not account for feedback effects. Additionally, the results are only optimal for level ground walking at 0.8 m/s. For an active device, this is less of an issue as control could vary with terrain, though for a fixed passive device, the assistance provided would vary with walking speed and environment. Finally, the subject pool provided by Hood et al. lacks diversity in both sex and reason for amputation. Differences in gait mechanics across a more varied group of users may illuminate further considerations for the device, such as which populations it is best suited for. Because of these limitations, we do not believe that the exact values reported by the optimization will be an immediate, ideal fit for any user. However, despite this, we assert the utility of this simulation as it informs future experiments. HIL optimization of devices, whether passive or active, is a time-intensive and fatiguing process–especially for individuals with gait difficulties. It is essential to make every possible effort to reduce the load on the subject, which is the primary benefit of these proposed methods. The short computational time means the method is apt for incorporation into human experiments or in a clinical setting. After gathering initial control data of joint angles and GRFs, this optimization could be completed during a rest period, after which the simulation-optimal configuration could be deployed and tested. We recognize that few clinics contain a fully equipped gait lab capable of collecting such data, though emerging methods of video-based motion capture and machine learning-based GRF prediction are quickly making these methods a feasible reality (Uhlrich et al., 2023; Tan et al., 2024).

Ideally, this informed starting point will reduce the number of HIL iterations necessary to determine the physically realized optimal configuration of the device. In future work, we hope to address these limitations, improving the quality of the simulation process to generate a better-informed starting point. Predictive simulation via OpenSim Moco could capture changes in kinematics caused by the device, for instance (Dembia et al., 2020). Parallel optimizations could be performed for a variety of terrains, and in the case of a passive device, we could generate a globally optimal device profile. The model of the device itself could be improved by including the nonlinear qualities of resistance bands and by modeling the interaction forces between the user and the wearable, as in Luo et al. (2024). Finally, as we move toward human subject trials, we hope to recruit from a wider variety of users to ensure robustness over a diverse user set.

Though even in their current standing, the results still inform us of the necessity of user-specific application and the failure of K-groups to distinguish results. We hypothesized there would be a high variability across subjects in each optimization parameter but that the separation into K2 and K3 groups would reduce this variability. Per the results, we only partially accept this hypothesis. The resting length of each band returned very low variability, less than 1 cm in all cases. This points toward the ideal resting length being less specific to the user. However, the stiffness of each band returned a high variance between subjects, as hypothesized. Separating these results into K2 and K3 groups, the variance did not consistently trend down. For some parameters, it decreased, though in others, it increased dramatically. There was not a consistent behavior for any one band, either of the K groups, or either optimization case. As we look toward human subject experiments, these results inform us that each individual user must be considered uniquely and that K-level does little to inform the assistance needs of any particular person.

Despite the absence of mobility-group level trends on device configurations, our post hoc analysis presented some promise for predicting optimal configuration from subject characteristics. The strongest and most significant relationship in both cases was that of default length of the band to resting length. This is admittedly expected, as the resting length must scale with the size of the device. Though, we note the relationship is not entirely linear, with variance here attributed to differences in user needs. We also observed a significant correlation between peak flexion moment and stiffness in Case 1. As the primary biological demand grows, the device must be stiffer to accommodate. Though, the correlation is weaker than expected and only significant independently in one band. This reflects a greater nuance in user needs and how the device is optimized to meet them. This relationship is further confirmed by the high variances in stiffnesses for Case 1 we see in Table 1. An equivalent relationship was not observed in Case 2 due to a difference in the optimization process. To meet the 1.5% goal in Case 1, the device needed to rely on force generation from both elements, whereas in Case 2, the device output was normalized, meaning no such requirement existed. As the results show, biasing heavily toward one band allowed for a higher quality fit, though across the 18 subjects, it seems largely random which of the two bands it chose to be the stiffer. For this reason, neither of the two stiffnesses is significantly related to the peak flexion moment. The final correlation of interest is that of peak abduction moment and stiffness. For a number of subjects, such as TF18 and TF15 in Figure 3, we observe a high abduction moment during stance, which the device has a limited capacity to compensate for. Only Band 1 is positioned to create an abduction moment, and even then, any significant imbalance would create a net rotational moment, which is undesirable. Despite this, we expected to see some correlation between peak abduction moment and stiffness in Band 1, as in these particular cases of a high abduction moment, the device should have valued compensating for some portion of the moment. This does not occur in either case; however, we see in both cases a significant inverse relationship between stiffness and peak abduction moment in Band 2 (i.e., for users with high peak abduction moments, the antagonistic band tends to be less stiff, resulting in a net abduction moment). As given in Table 2, there are several additional significant correlations. These relationships did not bear similar intuitive explanations, though nonetheless could be used to inform a fitting of the device. In short, the ideal resting length and stiffness of each band can be roughly informed by the default length of the device, the peak flexion moment, and the peak abduction moment. However, these correlations are generally weak and cannot be used to directly fit the device. This result further confirms the importance of subject-specific, simulation-based methods to inform HIL optimization.

**Table 1.** Mean and variance of optimization parameters as well as success metrics, separated by K group and optimizer configuration

			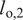 (m)	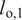 (m)	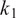	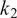	RMSE	MPE (%)	Comp. (%)	
Whole group	Case 1	Mean	0.562	0.595	123.785	135.112	0.277	159.764%	1.156%
		Variance	0.003	0.003	1757.939	934.431	0.023	—	0.000%
	Case 2	Mean	0.568	0.581	0.581	0.650	0.271	284.634%	—
		Variance	0.003	0.006	0.182	0.200	0.022	—	—
Within K2	Case 1	Mean	0.554	0.590	104.882	136.156	0.242	−23.927%	1.140%
		Variance	0.003	0.004	2611.009	1053.987	0.020	—	0.000%
	Case 2	Mean	0.560	0.564	0.482	0.774	0.238	12.898%	—
		Variance	0.003	0.009	0.179	0.171	0.020	—	—
Within K3	Case 1	Mean	0.570	0.600	142.687	134.068	0.312	343.454%	1.171%
		Variance	0.002	0.003	190.235	812.694	0.023	—	0.001%
	Case 2	Mean	0.577	0.601	0.680	0.525	0.305	556.371%	—
		Variance	0.003	0.002	0.166	0.198	0.023	—	—

*Note*: For properties, l_o_ is the resting length of each band and k is the stiffness. For Case 1, the stiffnesses are in N/m. For Case 2, stiffnesses are normalized and reported as a unitless value. Band 1 is the lateral band, and Band 2 is the medial band. RMSE and MPE are taken between the scaled biological moment profile and the device profile. Compensation is the maximum percentage of the biological flexion moment that the device achieves.

**Table 2.** The correlation and significance of each subject characteristic and design parameter

Case 1							
Subject characteristic	Design parameter	Band 1		Band 2		Combined	
		Correlation	*p*-value	Correlation	*p*-value	Correlation	*p*-value
Default length	*l*_o_	0.9478	**0.0000**	0.9579	**0.0000**	0.9112	**0.0000**
Weight	*l*_o_	0.5362	**0.0218**	0.5636	**0.0149**	0.5263	**0.0010**
Abd. moment	*l*_o_	−0.5145	**0.0289**	−0.5423	**0.0201**	−0.5058	**0.0016**
Add. angle	*l*_o_	0.5035	**0.0331**	0.5241	**0.0256**	0.4917	**0.0023**
Flex. moment	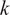	0.4437	0.0651	0.4800	**0.0438**	0.4482	**0.0061**
Abd. moment	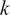	−0.5907	**0.0098**	0.1686	0.5036	−0.2642	0.1195
Flex. moment	*l*_o_	0.1699	0.5003	0.1320	0.6016	0.1435	0.4038
Ext. angle	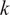	−0.1152	0.6491	0.3488	0.1560	0.0786	0.6487
Weight	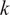	−0.0570	0.8221	−0.0205	0.9358	−0.0406	0.8140
Add. angle	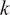	0.3283	0.1835	−0.4003	0.998	0.0206	0.9051
Ext. angle	*l*_o_	−0.0478	0.8506	0.0199	0.9377	−0.0119	0.9453
Default length	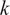	0.1322	0.6009	−0.1962	0.4352	−0.0061	0.9718

*Note*: Band 1 and Band 2 refer to within a single band, whereas combined considers both bands as a single dataset. The results are sorted by significance in the combined set, with significant (p ≤ 0.05) relationships in bold.

## 5. Conclusion

In this article, we develop and implement a simulation-based approach to optimize the behavior of wrapping hip flexion devices for the assistance of individuals with TFAs. We apply the method to 18 individuals with unilateral TFAs whose shape and level-ground gait are provided in the dataset of Hood et al. Of the individuals considered, 9 are of the K2 mobility level and 9 are of K3. The device is modeled via OpenSim wrapping surfaces, and we analyze the mechanics of the model for each subject during an average stride of level-ground walking at 0.8 m/s. In this work, we consider both passive devices made of soft elastic elements, as well as motor-driven cable-based devices. Reflecting this, we split the problem into optimizations over two cost functions. The optimization solves for the set of resting lengths and stiffnesses that generate a moment profile with the least squared difference to the prior biological moments of the hip via an interior-point algorithm. The problems are formulated and bounded to return results reasonable for either application. We hypothesized that the optimal device parameters would show a high variance between subjects but that the within-group (K2, K3) variances would be lower than the global values. We partially accept our hypothesis for both the active and passive optimizations as stiffnesses consistently returned high variances, and splitting into K groups reduced the variance of some parameters. However, the trends were not uniform as resting lengths all returned low variances, and some parameters had higher variances within groups. Given K group to be a poor indicator of device fit, we inspected the relationship between subject characteristics and optimal device parameters. We found significant relationships primarily with the default length of the device, the peak flexion moment, and the peak abduction moment; however, even these characteristics are not sufficient to fully predict the ideal fit. Broadly speaking, these results reflect the necessity to understand each user as an individual, regardless of K group or specific characteristics. These simulations exist to inform the ways in which we apply such devices in future HIL optimizations. Most importantly, we hope these methods will ease the physical load placed on users during HIL optimizations and allow users to more quickly arrive at an optimal configuration.

## Data Availability

The data used in this work is that of Hood et al. and can be found on Figshare: https://doi.org/10.6084/m9.figshare.c.4962305. The code that supports the findings of this study are available from the corresponding author, F.E., upon reasonable request.
